# Admission braden scale is an effective marker for predicting pneumonia in critically ill patients with traumatic brain injury

**DOI:** 10.1007/s10143-025-03571-5

**Published:** 2025-05-21

**Authors:** Xuelian Meng, Xia Li, Zhihong Tang

**Affiliations:** 1 Department of Critical Care Medicine, West China Hospital, Sichuan University, Chengdu, China; 2 West China School of Nursing, Sichuan University, Chengdu, China

**Keywords:** Traumatic brain injury, Pneumonia, Braden scale, Intensive care, Nomogram

## Abstract

Pneumonia is a common and serious complication among patients with traumatic brain injury (TBI), significantly impacting their prognosis. The Braden Scale (BS), a tool for assessing the risk of pressure ulcers, has been shown to effectively predict outcomes in various conditions. This study aims to evaluate the predictive value of the Braden Scale for TBI-related pneumonia and to develop a pneumonia prediction model for TBI patients in the intensive care unit (ICU). Clinical data of TBI patients who had a BS documented on ICU admission were retrospectively extracted from the Medical Information Mart for Intensive Care database. Restricted cubic spline (RCS) was employed to evaluate the nonlinear relationship between BS and pneumonia. Univariable and multivariable logistic analyses identified risk factors for TBI-related pneumonia, facilitating the construction of a nomogram model. The nomogram underwent internal validation through receiver operator characteristic (ROC) curve, calibration curve, and decision curve analysis. This study involved 2,175 TBI patients, among whom 516 (23.7%) with pneumonia. The total BS score and each subscale score of pneumonia patients were significantly lower than those of non-pneumonia patients (all *P* < 0.001). RCS analysis revealed a nonlinear L-shaped relationship between BS and the risk of pneumonia. Multivariable analysis identified eight risk factors for TBI-related pneumonia, including BS < 15, male, calcium, heart failure, chronic pulmonary disease, sepsis, respiratory, and temperature. By incorporating these variables, the predictive nomogram achieved an area under the ROC curve (AUC) of 0.803 (95% CI, 0.782–0.824) and had a well-fitted calibration curve and good clinical practicability. Furthermore, the predictive value of the nomogram surpasses that of common severity score, including the Glasgow Coma Scale (AUC = 0.530), Simplified Acute Physiology Score II (AUC = 0.588), Sequential Organ Failure Assessment (AUC = 0.610), and Acute Physiology Score III (AUC = 0.607). The admission BS is a simple and reliable indicator for predicting TBI-related pneumonia. Incorporating BS into predictive model may enhance the assessment of pneumonia risk in TBI patients, thereby facilitating the identification of high-risk populations and promoting timely intervention.

**Supplementary Information:**

The online version contains supplementary material available at 10.1007/s10143-025-03571-5.

## Introduction

Traumatic brain injury (TBI) is a common cause of permanent disability and death among patients. Globally, the annual incidence of TBI is estimated to range between 27 and 69 million, with at least half of the population likely to experience one or more TBIs in their lifetime [1, 2]. Survivors often face significant disabilities and mental health disorders, placing considerable burdens on families and society as a whole [3]. TBI patients frequently require prolonged bed rest and may necessitate tracheal intubation and mechanical ventilation due to consciousness disorders following trauma. Consequently, their risk of developing respiratory complications is markedly elevated compared to that of regular patients, with pneumonia being the most common complication observed [4]. It has been reported that approximately 20.4% to 61% of TBI patients may experience ventilator-associated pneumonia [5, 6]. The onset of pneumonia not only prolongs the duration of mechanical ventilation but also extends both the intensive care unit (ICU) and hospital stays, serving as an independent risk factor for poor outcomes [6]. Therefore, it is crucial to identify a reliable and effective scale for predicting the risk of pneumonia following TBI in order to facilitate early management. Several factors have been shown to be associated with the incidence of pneumonia in TBI patients include altered consciousness, age, alcohol abuse, sedation, chest trauma, and the prophylactic use of antibiotics [6–9]. For instance, TBI patients with disturbances in consciousness may experience aspiration and diminished cough reflexes, which can lead to increased sputum production and subsequent lung infections [10]. Additionally, the interaction between the brain and lung also plays a critical role in the pathophysiology of secondary lung diseases following TBI [11]. However, there is currently no established predictive model for pneumonia after TBI that adequately addresses these risk factors, and little is known about the relative importance of these variables in terms of their predictive value. The management of TBI-related pneumonia still remains a significant challenge.

The Braden Scale (BS) is a bedside nursing assessment tool originally designed to predict the risk of pressure ulcers [12]. It evaluates patients'skin integrity and overall health status based on six factors assessed at admission: sensory perception, mobility, activity level, moisture level, nutritional status, and friction/shear. The total score ranges from 6 to 23, with higher score indicating an increased risk of pressure ulcers. Due to its independence from laboratory data, this scale is both convenient and widely used in clinical practice [13]. Recent studies have indicated that the applicability of the BS extends beyond merely assessing pressure ulcer risk; it has been shown to effectively predict adverse clinical outcomes for various conditions including myocardial infarction, ischemic stroke, delirium, COVID-19, and cardiac patients in intensive care units [14–18]. Recently, a retrospective study involving 414 patients with acute ischemic stroke (AIS) demonstrated that BS may serve as an effective tool for predicting subsequently pneumonia [19]. The area under the receiver operating characteristic curve (AUC) of BS in forecasting post-AIS pneumonia was found to be 0.883, with a cutoff score set at 18 points. Furthermore, a multivariable analysis conducted on 629 patients with intracerebral hemorrhage revealed that lower BS scores were independently associated with stroke-associated pneumonia and exhibited moderate predictive value, evidenced by an AUC of 0.760 [20]. However, the relationship between BS and pneumonia after TBI has not been thoroughly investigated. In this context, we aimed to assess the feasibility of utilizing the BS to predict the risk of TBI-related pneumonia and to develop a predictive nomogram for pneumonia in ICU patients with TBI.

## Methods

### Data sources

This retrospective study was conducted using the Medical Information Mart for Intensive Care (MIMIC)-IV version 2.2 database. Overall, MIMIC-IV is a publicly accessible and free database that contains comprehensive information on 299,712 adult patients, which includes 431,231 inpatient admissions and 73,181 ICU admissions at Beth Israel Deaconess Medical Center in Boston, USA, spanning from 2008 to 2019 [21]. Our research team has successfully completed the online training course offered by the National Institutes of Health to gain authorization for utilizing this database. The establishment of this database received approval from the Institutional Review Boards of both the Massachusetts Institute of Technology and Beth Israel Deaconess Medical Center [22]. Importantly, all patient health information within this database has been anonymized, which alleviates the need for further ethical approval applications.

### Patient selection

The study includes ICU patients admitted with a primary diagnosis of TBI and related conditions, including concussions, brain contusions, traumatic intracranial hemorrhages, and skull fractures. To identify TBI patients in the MIMIC-IV database, we utilized the International Classification of Diseases 9 th Edition (ICD-9) and 10 th Edition (ICD-10) coding standards. The diagnosis for TBI patients was determined based on ICD-9 codes within 85, 800, 801, 803, 804, or ICD-10 codes within S06 [23, 24]. The study population consisted of adult patients aged 18 years or older who were admitted to the ICU for the first time between the years 2008 and 2019 as recorded in the database. Patients without a BS score documented on ICU admission or those with an ICU stay shorter than 24 h were excluded from the analysis. After screening, a total of 2,175 patients were ultimately confirmed for inclusion in this study.

**Fig. 1:**
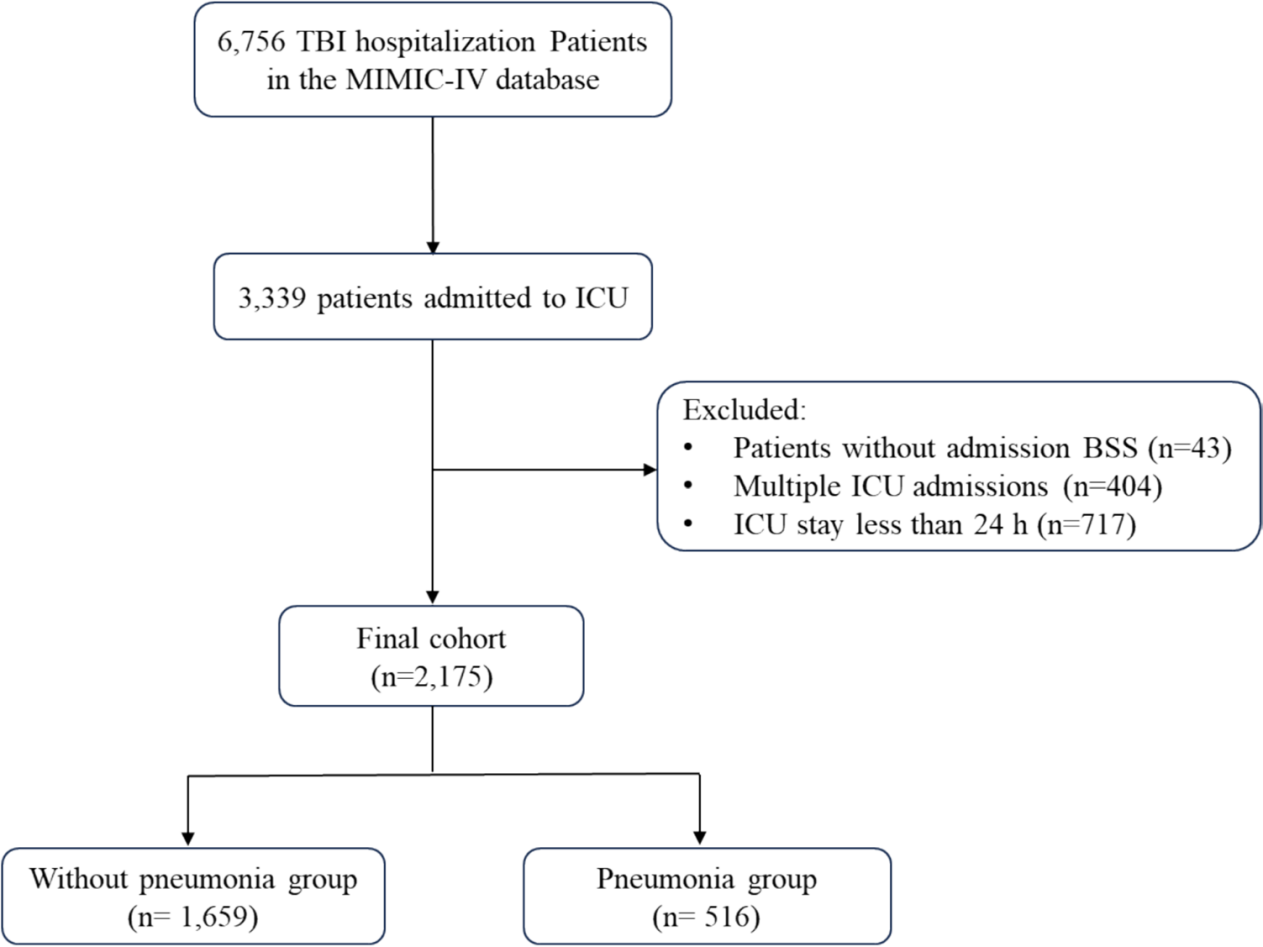
The flowchart of this study. TBI traumatic brain injury, MIMIC-IV Medical Information Mart for Intensive Care-IV, ICU intensive care unit, BSS Braden Scale Score

To enhance the generalizability of our findings, we did not distinguish between hospital-acquired pneumonia (HAP) and ventilator-associated pneumonia (VAP). Pneumonia identification in the MIMIC-IV database was based on ICD-9 and ICD-10 coding, covering all related diagnoses, including"pneumonia","hospital-acquired pneumonia","ventilator-associated pneumonia", and"pneumonia caused by XX bacteria"[25–27]. We further classified TBI patients based on the presence or absence of pneumonia. A detailed inclusion process is illustrated in Fig. 1.

### Braden scale

In this study, the BS score documented at the time of ICU admission were collected. The Braden Scale is a standardized, evidence-based assessment tool widely utilized in clinical practice to evaluate and document patients'risk of developing pressure ulcers [12]. This score consists of six subscale factors, and each factor is assigned a value ranging from 1 to 4. Notably, the factor friction/shear is scored between 1 and 3 as an exception. The scores across six subscales are summed, and the total score is then used to indicate the patient's risk level for developing pressure ulcers: (1) Mild risk: 15–18; (2) Moderate risk: 13–14; (3) High risk: 10–12; (4) Severe risk: less than 9 [28]. The detailed specifications of the BS can be found in supplementary Fig. 1.

### Data collection

The structured query language with PostgreSQL (version 15.2) was employed to extract the following study variables: demographic characteristics, comorbidities upon admission, severity scores, admission BS scores, vital signs, and laboratory indicators recorded during the first 24 h following ICU admission. In instances where vital signs or laboratory variables were measured multiple times, we selected the value that reflected the highest severity of illness. Additionally, we documented interventions during the ICU stay, including mechanical ventilation, renal replacement therapy, and vasopressor administration. Variables with over 10% missing data were excluded, and remaining missing values were imputed using the “MICE” package in R. Details are shown in Supplementary Fig. 2.

### Statistical analysis

Patients with TBI were categorized into pneumonia and non-pneumonia groups. Continuous variables exhibiting a normal distribution were described using mean ± standard deviation, while those with a non-normal distribution were summarized using median values. To evaluate differences between the pneumonia and non-pneumonia groups, we employed Pearson's χ^2^ test for categorical variables, Student's t-test for normally distributed continuous variables, and the Mann–Whitney U test for non-normally distributed continuous variables. To evaluate the relationship between BS and the risk of pneumonia, we used univariable restricted cubic spline (RCS) regression analysis to identify potential nonlinear associations. Based on the cut-off points derived from the RCS, we categorized the continuous BS scores into binary factors, utilizing the higher BS value as a reference for subsequent analysis. Logistic regression analysis was conducted to assess the relationship between the BS and pneumonia. In univariable analysis, variables associated with pneumonia that yielded a P value of less than 0.05 were included in the multivariable analysis. To avoid model overflow due to multicollinearity, variance inflation factors (VIFs) were calculated and any variables with a VIF greater than 2.5 was excluded from further consideration [29]. Subsequently, the screened factors were integrated by multivariable logistic analysis, and the Akaike Information Criterion (AIC) was employed as the stopping rule, ensuring that the final model achieved a minimized AIC value while retaining the fewest number of variables [30]. A nomogram predictive model was constructed based on the multivariable analysis. Its performance was internally validated using the bootstrap method with 1000 repetitions, employing Receiver Operating Characteristic (ROC) curve analysis, calibration curve analysis, and Decision Curve Analysis (DCA). Finally, the models with and without BS were further evaluated through net reclassification improvement and integrated discrimination improvement.

All statistical analyses were performed using the R software (version 3.6.0). The following R packages were utilized: “autoReg”, “rms”, “ggrcs”, “rmda”, “fbroc”, and “PredictABEL”. P values less than 0.05 (two-sided test) were considered statistically significant.

## Results

### Clinical characteristics of patients

This study included 2,175 patients with TBI, with a median age of 65.85 years. Within this cohort, a total of 516 (23.7%) patients developed pneumonia after TBI. A comparison of baseline characteristics between the pneumonia group and the non-pneumonia group is presented in Table 1. The pneumonia patients exhibited a higher proportion of males and was more likely to have comorbidities such as chronic lung disease, heart failure, diabetes, and sepsis. Additionally, these individuals presented with lower BS and higher severity scores, alongside more pronounced abnormalities in vital signs and laboratory findings. During hospitalization, patients with TBI-related pneumonia had higher rates of mechanical ventilation, tracheostomy, vasopressor use, and dialysis treatment. Furthermore, those who developed pneumonia had longer ICU and overall hospital stays compared to those without pneumonia. Their hospital mortality was significantly higher, but there was no obvious difference in ICU mortality between the two groups.

**Table 1:** Baseline characteristics of participants

Variables	Overall (*n* = 2,175)	Non-pneumonia (*n* = 1,659)	Pneumonia (*n* = 516)	*P* value
Age (years)	65.85 (47.61, 80.96)	66.20 (47.93, 81.55)	65.29 (45.84, 79.65)	0.14
Male (%)	1,376 (63.3%)	1,011 (60.9%)	365 (70.7%)	< 0.001
Ethnicity (%)				0.005
White	1,350 (62.1%)	1,066 (64.3%)	284 (55.0%)	
Others	825 (37.9%)	593 (35.7%)	232 (45.0%)	
Comorbidities (%)				
Chronic pulmonary disease	248 (11.4%)	168 (10.1%)	80 (15.5%)	< 0.001
Hypertension	892 (41.0%)	679 (40.9%)	213 (41.3%)	0.9
Heart failure	258 (11.9%)	174 (10.5%)	84 (16.3%)	< 0.001
Diabetes	415 (19.1%)	300 (18.1%)	115 (22.3%)	< 0.001
Renal disease	223 (10.3%)	163 (9.8%)	60 (11.6%)	0.2
Sepsis	981 (45.1%)	569 (34.3%)	412 (79.8%)	< 0.001
Vital signs				
Heart rate (min)	81.68 (70.98, 93.24)	80.11 (70.05, 91.74)	85.38 (76.33, 97.16)	< 0.001
SBP (mmHg)	124.50 (114.96, 133.51)	124.60 (115.43, 133.60)	124.14 (113.34, 133.41)	0.4
DBP (mmHg)	64.80 (58.14, 72.37)	65.13 (58.29, 72.48)	63.61 (57.57, 71.72)	0.051
Respiratory rate (min)	17.98 (16.31, 20.08)	17.69 (16.13, 19.58)	18.99 (17.12, 21.42)	< 0.001
Body Temperature (℃)	37.00 (36.75, 37.35)	36.95 (36.73, 37.25)	37.20 (36.85, 37.60)	< 0.001
SPO_2_ (%)	97.75 (96.35, 99.00)	97.60 (96.28, 98.84)	98.31 (96.74, 99.41)	< 0.001
Laboratory indicators				
WBC (10^9^/L)	10.95 (8.35, 14.17)	10.70 (8.05, 13.70)	12.10 (9.30, 15.50)	< 0.001
Platelet (10^9^/L)	197.00 (155.00, 245.50)	198.00 (158.00, 246.50)	190.00 (143.75, 237.75)	0.009
Hemoglobin(10^12^/L)	11.75 (10.40, 13.05)	11.85 (10.45, 13.15)	11.55 (10.15, 12.75)	< 0.001
Bicarbonate (mmol/L)	23.50 (21.50, 25.50)	23.50 (21.50, 25.50)	23.00 (20.50, 25.00)	< 0.001
Sodium (mmol/L)	139.50 (137.00, 142.00)	139.50 (137.00, 141.50)	140.00 (137.00, 142.00)	0.079
Potassium (mmol/L)	4.00 (3.75, 4.40)	4.00 (3.75, 4.40)	4.05 (3.80, 4.40)	0.2
Creatinine (mg/dL)	0.85 (0.70, 1.10)	0.85 (0.70, 1.10)	0.90 (0.70, 1.15)	0.015
BUN (mg/dL)	15.50 (11.00, 21.00)	15.00 (11.00, 21.00)	16.00 (12.00, 22.50)	0.004
Calcium (mmol/L)	8.50 (8.00, 8.90)	8.55 (8.10, 8.95)	8.30 (7.85, 8.70)	< 0.001
Chloride (mmol/L)	104.50 (101.50, 107.50)	104.00 (101.00, 107.00)	105.00 (102.00, 108.00)	< 0.001
Glucose (mmol/L)	128.50 (108.50, 154.25)	125.50 (107.00, 150.00)	139.00 (117.00, 163.50)	< 0.001
Prothrombin time (s)	12.65 (11.70, 14.00)	12.65 (11.65, 13.85)	12.80 (11.90, 14.40)	0.002
PTT (s)	27.60 (25.45, 30.50)	27.60 (25.50, 30.40)	27.60 (25.29, 30.86)	> 0.9
Scoring systems				
BS	15.00 (13.00, 17.00)	15.00 (13.00, 17.00)	14.00 (12.00, 15.00)	< 0.001
GCS	14.00 (12.00, 15.00)	14.00 (13.00, 15.00)	15.00 (11.00, 15.00)	0.035
SOFA	3.00 (2.00, 5.00)	3.00 (2.00, 4.00)	4.00 (2.00, 6.00)	< 0.001
SAPSII	31.00 (24.00, 39.00)	31.00 (23.00, 38.00)	34.00 (27.00, 42.25)	< 0.001
APSIII	35.00 (27.00, 46.00)	34.00 (26.00, 44.00)	40.00 (30.00, 53.00)	< 0.001
ICU intervention				
Vasopressor use (%)	420 (19.3%)	239 (14.4%)	181 (35.1%)	< 0.001
MV (%)	990 (45.5%)	603 (36.3%)	387 (75.0%)	< 0.001
tracheostomy	137 (6.3%)	30 (1.8%)	107 (20.7%)	< 0.001
RRT use (%)	48 (2.2%)	27 (1.6%)	21 (4.1%)	< 0.001
ICU-stay (days)	2.79 (1.72, 5.69)	2.31 (1.61, 3.87)	8.15 (3.67, 14.54)	< 0.001
Hospital-stay (days)	7.31 (4.20, 13.49)	5.83 (3.73, 9.65)	16.56 (9.82, 26.02)	< 0.001
ICU mortality (%)	148 (6.8%)	109 (6.6%)	39 (7.6%)	0.4
Hospital mortality (%)	288 (13.2%)	203 (12.2%)	85 (16.5%)	0.013

Data is presented in numerical form with percentages in parentheses, or as medians with interquartile ranges noted in parentheses. *APSIII* Acute Physiology Score III, *BS* Braden Scale, *BUN* blood urea nitrogen, *DBP* diastolic blood pressure, *GCS* Glasgow Coma Score, *ICU* intensive care unit, *MV* mechanical ventilation, *PTT* partial thromboplastin time, *RRT* renal replacement therapy, *SAPSII* Simplified Acute Physiology Score II, *SBP* systolic blood pressure, *SOFA* Sequential Organ Failure Assessment, *SpO*_*2*_ oxyhemoglobin saturation, *WBC* white blood cell

### Distribution of admission BS score and subscale score

**Fig. 2:**
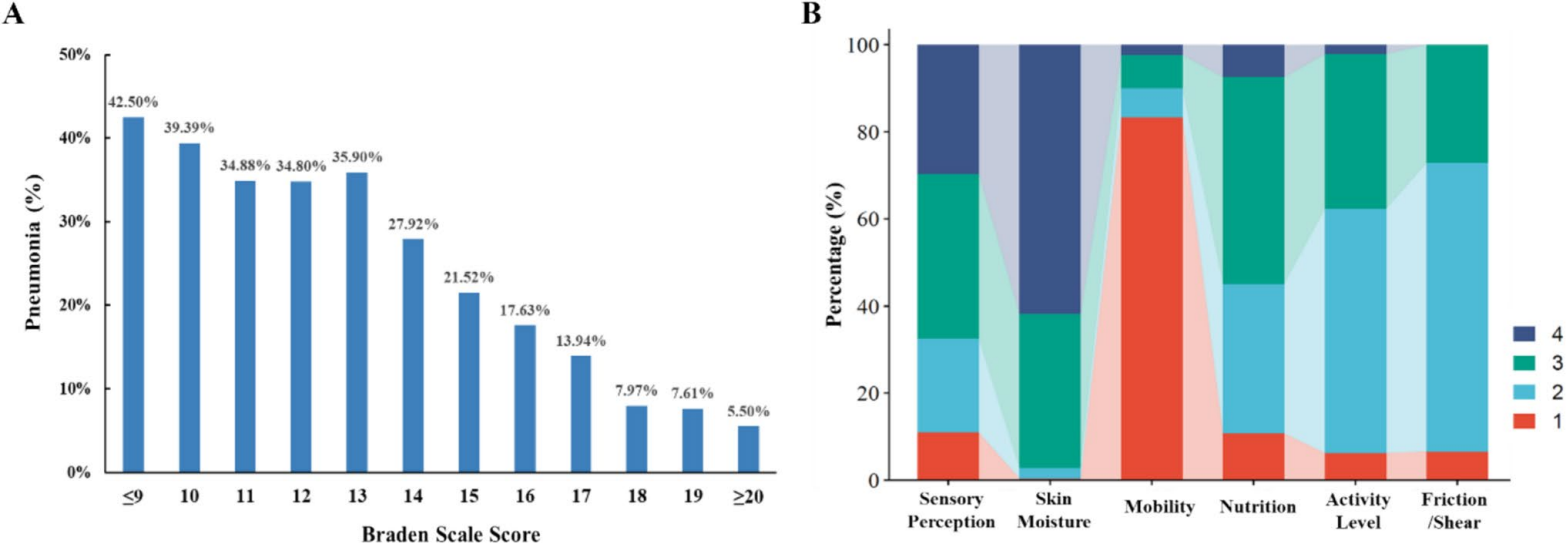
Distribution of BS score and subscale score. **A** The incidence of pneumonia in patients with different BS score. **B** Distribution of individual Braden subscale scores within the study population. BS, Braden Scale

As the admission BS score increased, the incidence of pneumonia in TBI patients gradually rose **(**Fig. 2A**)**. Notably, when the BS score ≥ 20, only 5.5% of TBI patients developed pneumonia; conversely, when the BS score was ≤ 9, as many as 42.5% of TBI patients exhibited pneumonia. This finding suggested a significant correlation between lower BS score and an elevated risk of pneumonia. The distribution of admission BS subscale score within the population was illustrated in Fig. 2B. Among the various BS subscale scores, a majority of patients (> 60%) demonstrated normal (4 points) or slightly limited (3 points) values for Sensory Perception and Skin Moisture. In contrast, most patients exhibited either severely abnormal (1 point) or significantly restricted scores (2 points) in Activity Level and Friction/Shear. Notably, nearly 80% of TBI patients presented with a completely limited assessment of Mobility during their stay in the ICU.

### Association between BS score/subscale score and TBI-related pneumonia

**Fig. 3:**
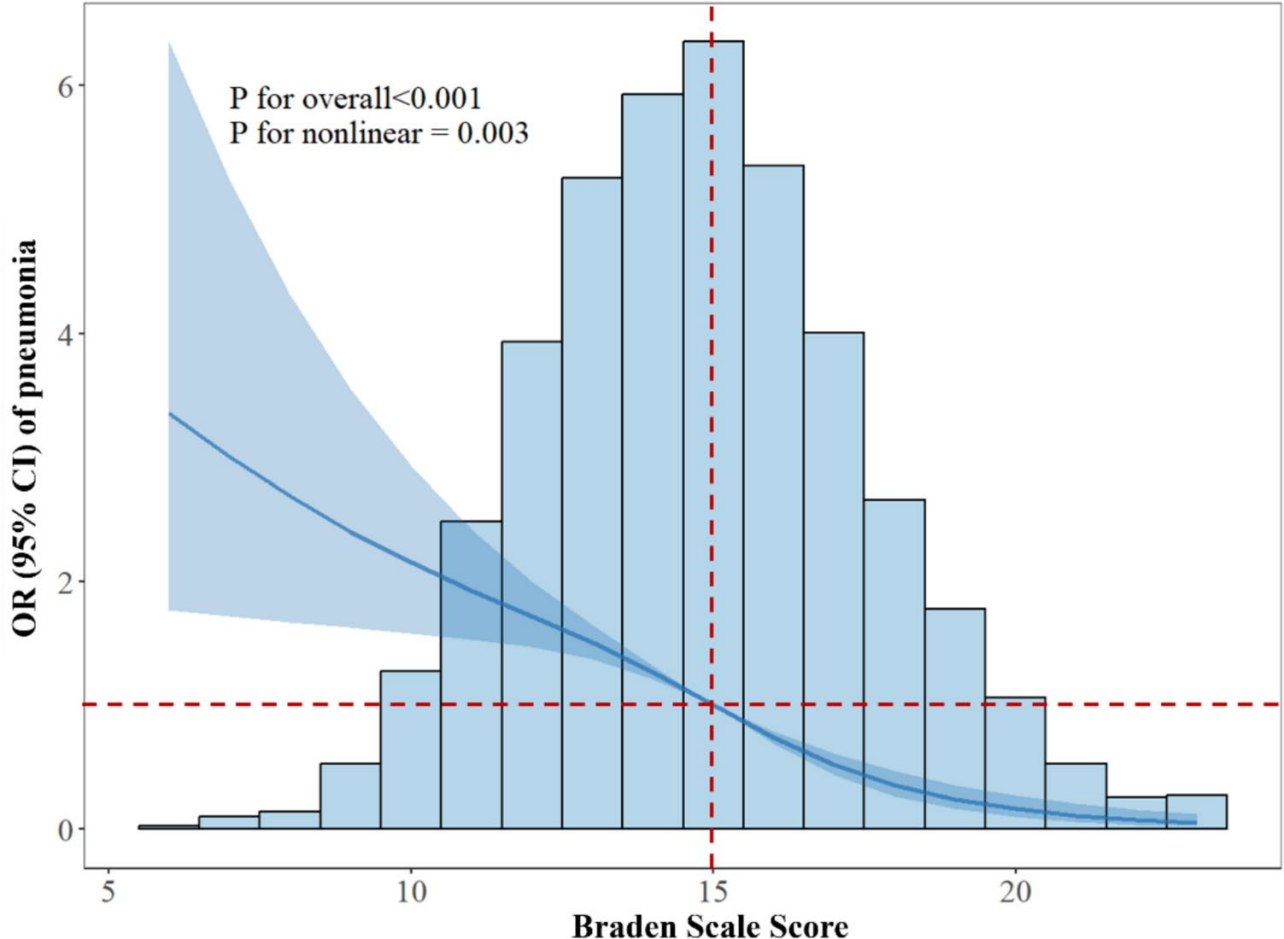
The RCS analysis of the relationship between BS and pneumonia risk in patients with traumatic brain injury. The intersection of the horizontal and vertical dashed lines indicated the BS value at which the odds ratio equal 1.0; therefore, a cutoff value for the BS score was established at 15. BS Braden Scale, OR odds ratio, CI confidence interval, RCS restricted cubic spline

Table 2 showed the distribution of admission Braden subscale scores among the pneumonia and non-pneumonia groups. TBI patients in the pneumonia groups demonstrated significantly lower subscale scores when compared to those without pneumonia (all *P* < 0.05). The univariable RCS regression demonstrated a nonlinear L-shaped relationship between continuous BS scores and the risk of TBI-related pneumonia (*P* for overall < 0.001, non-linear *P* value = 0.003), as shown in Fig. 3. The result of the multivariable logistic regression analysis with the minimum AIC value was shown in Table 3. The variables with a significance level of *P* < 0.05 included BS, male calcium, heart failure, chronic pulmonary disease, sepsis, respiratory rate, and body temperature. Of note, lower BS was significantly associated with an increased risk of TBI-related pneumonia (OR: 1.81 95% CI: 1.43–2.29, *P* < 0.001). There was no multicollinearity among these risk factors ultimately incorporated into the model (Supplementary Table 1).

**Table 2:** Braden subscale scores for the two groups

	Non-pneumonia (*n* = 1,659)	Pneumonia (*n* = 516)	*P* value
Sensory Perception	2.97 ± 0.95	2.51 ± 0.94	< 0.001
Activity	3.60 ± 0.55	3.52 ± 0.60	0.007
Mobility	1.34 ± 0.76	1.12 ± 0.45	< 0.001
Skin Moisture	2.61 ± 0.77	2.23 ± 0.76	< 0.001
Nutrition	2.39 ± 0.63	2.16 ± 0.59	< 0.001
Friction/Shear	2.45 ± 0.55	2.08 ± 0.50	< 0.001

Data were expressed as mean ± standard deviation

**Table 3:** Risk factors in the final predictive model

	OR (95% CI)	*P* value
BS < 15	1.81 (1.43, 2.29)	< 0.001
Male	1.38 (1.08, 1.75)	0.009
Calcium	0.82 (0.70, 0.90)	0.018
Heart failure	1.67 (1.19, 1.23)	0.003
Chronic pulmonary disease	1.82 (1.29, 2.57)	< 0.001
Sepsis	5.77 (4.51, 7.44)	< 0.001
Respiratory rate	1.11 (1.07, 1.15)	< 0.001
Body Temperature	1.54 (1.25, 1.90)	< 0.001

*BS* Braden Scale, *OR* Odds Ratio, *CI* Confidence Interval

### Construction and validation the nomogram

**Fig. 4:**
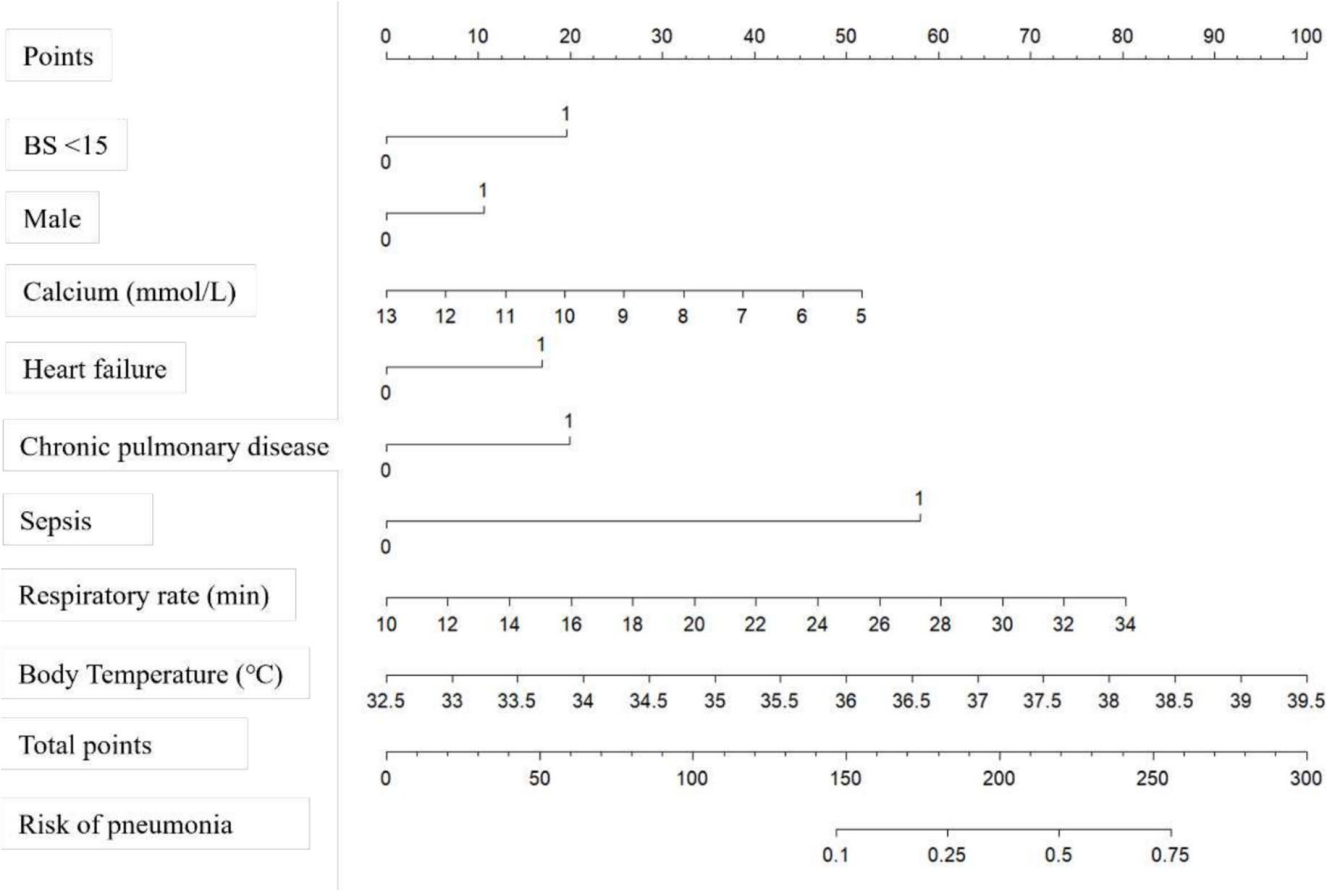
Nomogram based on Braden Scale for predicting pneumonia in traumatic brain injury patients. BS Braden Scale

**Fig. 5:**
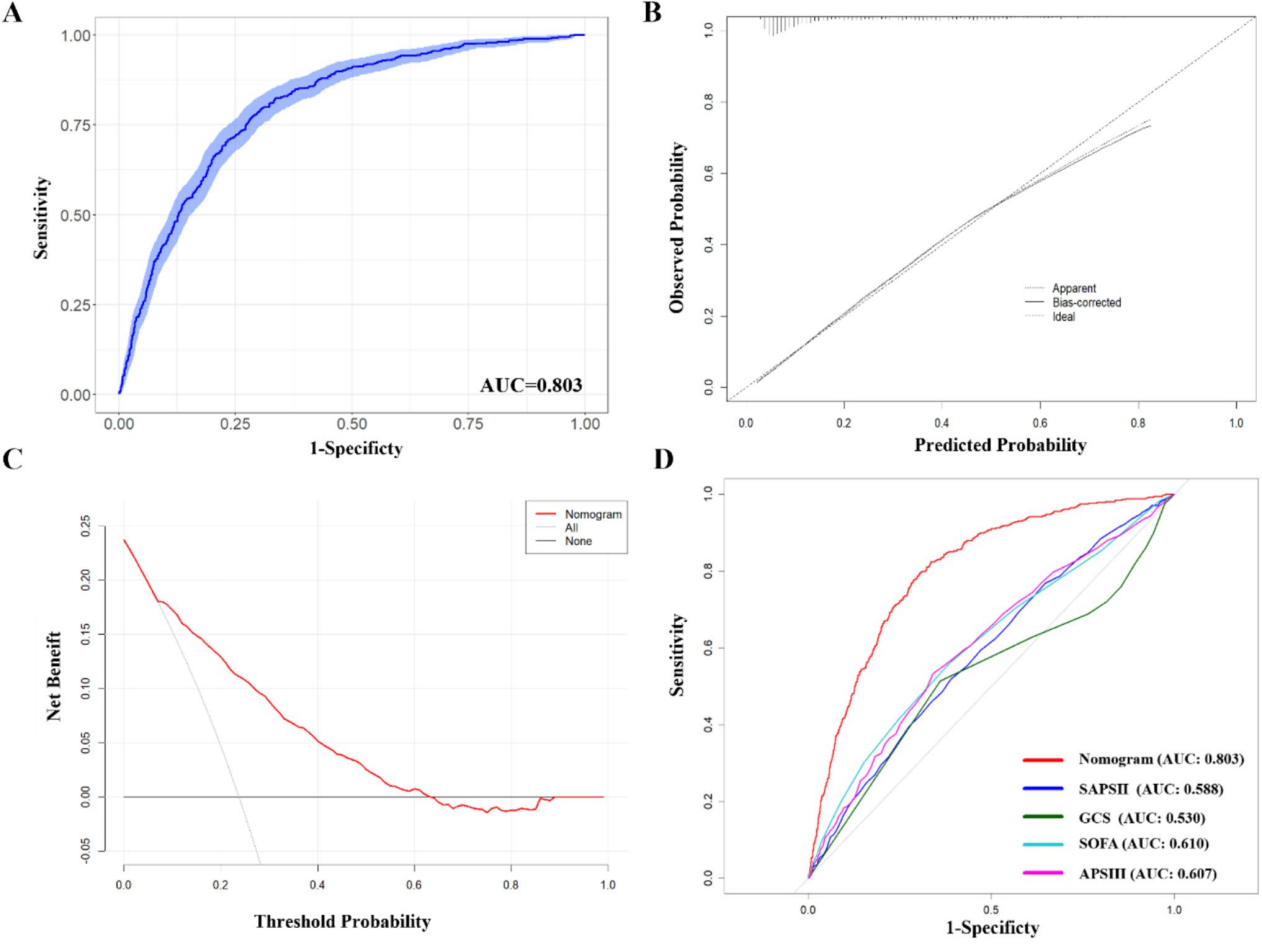
Performance of the nomogram. **A** The receiver operating characteristic curve (bootstrap 1000 repetitions), the bootstrap-corrected AUC is 0.803 (95% CI: 0.781–0.823). **B** Calibration plot (bootstrap 1000 repetitions). **C** Decision curve analysis. **D** The ROC curves of the nomogram and other severity scores. AUC area under the receiver operating characteristic curve, APSIII Acute Physiology Score III, GCS Glasgow Coma Scale, SAPSII Simplified Acute Physiology Score II, SOFA Sequential Organ Failure Assessment

The eight risk factors identified through multivariable analysis were employed to develop a nomogram prediction model for pneumonia in TBI patients, as illustrated in Fig. 4. The predictive nomogram achieved an AUC of 0.803 (95% CI, 0.782–0.824). Additionally, the bootstrap-corrected AUC, determined through 1000 repetitions, was found to be 0.803 (95% CI: 0.781–0.823), indicating that the model possessed strong predictive discrimination (Fig. 5A). The calibration curve (Fig. 5B) revealed a high degree of consistency between predicted values and actual observed outcomes within the patient population. The net benefit derived from the application of the nomogram was illustrated through a DCA curve as depicted in Fig. 5C. Furthermore, in comparison to other common severity scores such as the Glasgow Coma Scale (AUC = 0.530), Simplified Acute Physiology Score II (AUC = 0.588), Sequential Organ Failure Assessment (AUC = 0.610), and Acute Physiology Score III (AUC = 0.607), the nomogram demonstrated better predictive performance **(**Fig. 5D**)**. Comparative evaluation demonstrated that BS-added models achieved enhanced predictive performance, showing an increase in AUC alongside significant improvements in both net reclassification and integrated discrimination, thereby validating the better prognostic value of BS inclusion (Supplementary Table 2).

## Discussion

It is widely recognized that individuals with TBI may experience brain damage, altered consciousness, and prolonged bed rest [3]. These factors can impair airway protective mechanisms, weakening the cough reflex and reducing secretion clearance, which increases the risk of aspiration or micro-aspiration [4]. Consequently, there is a significantly higher incidence of pneumonia among TBI patients compared to regular ICU patients, adversely impacting their quality of life and survival [11]. Our study indicated that 23.7% of TBI patients developed pneumonia, which was comparable to the 20.4% reported by Robba et al. concerning TBI patients who progressed to VAP [6]. However, since we did not differentiate between VAP and HAP, the overall incidence of pneumonia appears relatively higher. Notably, the subgroup of TBI patients without concurrent pneumonia demonstrated higher admission GCS scores and shorter ICU stays, suggesting this population primarily comprised mild-to-moderate TBI injury cases. Nevertheless, these patients were admitted to ICU due to advanced age, comorbidities, and the need for neurological monitoring, reflecting the complexity of ICU admission decisions in clinical practice. Early identification of pneumonia risk is crucial for managing pulmonary complications in TBI patients [31]. Those assessed as low-risk should be closely monitored, while high-risk patients require proactive interventions, such as more frequent secretion drainage and timely antibiotic administration [32]. This highlights the critical need for the development of clinically convenient predictive indicators and applicable predictive models to evaluate the risk of TBI-related pneumonia within the ICU.

The BS is an essential tool for assessing the risk of pressure ulcers in patients, developed by Braden and Bergstrom in 1987 [12]. This scale can be assessed upon patient admission without the need for additional laboratory tests, which renders it both convenient and efficient for clinical application. In recent years, researches have shown that the BS is not only valuable for pressure ulcer risk assessment but also closely linked to disease prognosis. Lower BS scores have been associated with increased mortality in various conditions, including patients in coronary care units, those with myocardial infarction, COVID-19, cerebral infarction, and frailty [14, 15, 17, 18, 33, 34]. Furthermore, lower BS scores were also linked to the occurrence of specific diseases. Li et al. conducted a study on patients with acute coronary syndrome and found that BS scores were closely correlated with the incidence of acute kidney injury (AKI), suggesting its utility as a tool for identifying and preventing high-risk AKI cases [35]. A retrospective study involving 24,123 elderly critically ill patients revealed a significant negative correlation between BS scores upon ICU admission and delirium prevalence [16]. This suggests that the simplicity of the BS may reflect the overall health status and frailty in older patients [16]. Another study further confirmed that mortality differences due to frailty were more pronounced in individuals with higher BS scores [34]. Recent studies have indicated that the presence of BS may be linked to the incidence of pneumonia among general ward populations suffering from cerebrovascular diseases. One study involving 414 acute ischemic stroke (AIS) patients found that a cutoff score of 18 on the BS could serve as an effective clinical rating scale for predicting pneumonia following AIS, with an AUC of 0.883 [19]. Another study focusing on 629 cases of intracerebral hemorrhage indicated that lower BS scores were independent risk factors for pneumonia (OR 0.696; 95% CI 0.631–0.768) [20]. To our knowledge, this study was the first to explore the relationship between BS and pneumonia after TBI within the ICU. Our results showed that higher admission BS scores were associated with a lower incidence of TBI-related pneumonia, demonstrating a non-linear L-shaped relationship. Significant differences in BS subscale scores were also found between pneumonia and non-pneumonia groups. Furthermore, by employing a cutoff score of 15, individuals with high BS scores exhibited a significantly reduced risk of pneumonia compared to those with low BS scores. These findings suggested that the BS may serve as a convenient and effective biomarker for predicting the risk of pneumonia in TBI patients. The BS score could facilitate the identification of patients at high risk for TBI-related pneumonia, enabling early intervention aimed at improving their prognosis.

Given the significant predictive value of the BS and its ease of application in clinical settings, incorporating this scale into the management of pneumonia following TBI may yield substantial benefits. Through univariable and multivariable logistic regression analysis, we identified eight independent risk factors for the development of pneumonia in TBI patients: lower BS, male, decreased calcium, concurrent heart failure, chronic pulmonary disease, sepsis, as well as elevated body temperature and respiratory rate. These variables were consistent with some of the risk factors findings from previous studies [6–8]. It is noteworthy that Geng et al. employed lasso regression and logistic regression to identify variables such as temperature, sex, and chronic lung disease as predictors in the development of a pneumonia prediction model for ICU patients with TBI [36]. These variables correspond with several risk factors identified in our study; however, the emergence of additional differing predictors may be attributed to variations in statistical methodologies and parameter settings across different studies. Some studies have predicted the risk of VAP after TBI [37], while our research focuses more broadly on pneumonia caused by TBI, encompassing a wider population. Additionally, our findings reveal the correlation between BS and TBI-related pneumonia, as well as its feasibility in predictive applications. Our predictive model has several advantages. First, all predictive variables are available upon admission; while serum calcium requires laboratory testing, it is easily accessible in clinical practice, enhancing the model's applicability. Previous studies have attempted to develop models for pneumonia after TBI, but these often rely on imaging data or additional laboratory tests, which limits their applicability [38, 39]. Second, following internal validation, our model exhibited both a high discriminative ability and strong clinical utility, effectively identifying patients at high risk for pneumonia. Notably, our model exhibited superior predictive performance, as evidenced by significantly higher AUC values in comparison to the GCS, SAPS II, SOFA, and APS III. These scores are currently widely utilized in the ICU to assess the prognosis of patients with TBI [40]. In future prospective studies, we would further collect data on pneumonia scoring within the ICU to enhance the robustness of this research. Finally, we innovatively incorporated the BS into the TBI-pneumonia prediction framework, taking into account both the accessibility and its causal relationship with pneumonia. This offered compelling evidence for the utilization of the BS as a biomarker in the management of pneumonia in ICU TBI patients.

Although the core design of the BS is intended to assess the risk of pressure ulcers, its six subscales are all pathophysiologically linked to pulmonary complications in TBI patients. For instance, low scores on activity and mobility (≤ 3) indicate that patients may require prolonged bed rest, which increases the risk of pulmonary secretions retention; similarly, poor nutritional perception (nutritional intake score ≤ 2) is associated with immunosuppression and aspiration risks. Furthermore, moisture scoring reflects factors such as the effectiveness of respiratory secretion management—all contributing to an increased risk of lung infections. Additionally, previous studies have shown that BS scores correlate with pneumonia occurrence in ischemic stroke and intracerebral hemorrhage patients. And our study further corroborated that a lower admission BS score was associated with elevated risk of TBI-related pneumonia. Currently, the brain-lung interaction in TBI patients is receiving increasing attention [11]. Compared to general ICU patients, critically ill patients with TBI exhibited a significantly higher risk of complications, with pulmonary diseases being the most common [4]. Shortly after a brain injury, clinical pre-phase lung damage may occur, typically marked by abnormal respiratory mechanics and hypoxemia [41]. While the underlying mechanisms are not fully understood, factors such as neurogenic pulmonary edema, inflammatory responses, neurotransmitter-related damage, and side effects of neuroprotective treatments are considered key elements; these have been further examined within"blast injury"theory or"double-hit"models [11, 42]. When TBI leads to a sudden increase in intracranial pressure, sympathetic nervous system storms trigger a massive release of catecholamines, causing an immediate rise in intravascular pressure. This surge can rupture the alveolar-capillary membrane and lead to pulmonary edema. Concurrently, intracranial inflammatory responses from brain injury leads to excessive production of pro-inflammatory cytokines, such as interleukins and tumor necrosis factor, from damaged tissue. Altered blood–brain barrier permeability allows these cytokines into peripheral circulation, triggering a systemic inflammatory response. These inflammatory mediators are known as the “first hit”. Subsequent therapeutic measures for TBI—such as mechanical ventilation or surgery—and complications like pneumonia contribute to the “second hit”. Together, they create the “double-hit” model [11]. The BS provides an effective biomarker for early identification of patients at high risk for pneumonia. The predictive model based on this scale, tailored for pneumonia risk, is poised to enhance clinical management and facilitate proactive intervention for TBI patients.

This study has several limitations. First, the retrospective nature of this analysis may introduce inherent biases. Certain information in the MIMIC database could not be collected, such as patients'smoking and alcohol consumption histories; these factors may also serve as risk factors for pneumonia. Additionally, accurately defining the causal relationship between mechanical ventilation or tracheostomy treatment and pneumonia occurrence was not feasible. As a result, these confounding variables were excluded from the risk factor analysis, possibly omitting potential predictors [8]. Second, due to the limitations imposed by the inclusion criteria, this predictive nomogram may not be applicable to other populations—especially TBI patients who did not receive intensive care. Although the BS demonstrated predictive validity in ICU settings, its subscale sensitivity—particularly mobility assessments in sedated patients—may require context-specific interpretation. Furthermore, while real-world applicability was strengthened through our clinical prioritization, the predominance of mild-to-moderate TBI cases necessitates cautious generalization. Future studies should prioritize validating critical thresholds in severe TBI populations to establish clinical confidence. Finally, while the nomogram exhibited strong performance during internal validation, it still required external validation. Consequently, future research should be designed to include rigorous prospective studies aimed at further evaluating the applicability and value of this model.

## Conclusions

Our research findings show that a lower admission BS score was associated with elevated risk of pneumonia, and BS < 15 was an independent risk factor for pneumonia in TBI patients. The BS has proven to be a straightforward yet valuable indicator for predicting TBI-related pneumonia. The nomogram model that incorporates the admission BS could accurately forecast the incidence of pneumonia, and internal validation has confirmed both its robustness and performance. This model assists in stratifying and predicting the risk of pneumonia among TBI patients. We recommend conducting further prospective studies to validate the predictive value of this nomogram in clinical practice.

## Supplementary Information

Below is the link to the electronic supplementary material.

Supplementary file1 (DOCX 625 KB)

## Data Availability

Publicly available datasets were analyzed in this study. This data can be found here: https://mimic.mit.edu.

## References

[1] Dewan MC et al (2019) Estimating the global incidence of traumatic brain injury. J Neurosurg 130(4):1080–1097 29701556 10.3171/2017.10.JNS17352

[2] Injury GB (2019) Global, regional, and national burden of traumatic brain injury and spinal cord injury, 1990–2016: a systematic analysis for the global burden of disease study 2016. Lancet Neurol. 18(1):56–87 30497965 10.1016/S1474-4422(18)30415-0 PMC6291456

[3] Trieu C et al (2023) Overview of Hypothermia, Its Role in Neuroprotection, and the Application of Prophylactic Hypothermia in Traumatic Brain Injury. Anesth Analg 137(5):953–962 37115720 10.1213/ANE.0000000000006503

[4] Sharma R et al (2019) Infections after a traumatic brain injury: The complex interplay between the immune and neurological systems. Brain Behav Immun 79:63–74 31029794 10.1016/j.bbi.2019.04.034

[5] Esnault P et al (2017) Early-onset ventilator-associated pneumonia in patients with severe traumatic brain injury: incidence, risk factors, and consequences in cerebral oxygenation and outcome. Neurocrit Care 27(2):187–198 28432539 10.1007/s12028-017-0397-4

[6] Robba C et al (2020) Incidence, risk factors, and effects on outcome of ventilator-associated pneumonia in patients with traumatic brain injury: analysis of a large, multicenter, prospective. Observation Longitud Stud Chest 158(6):2292–2303 10.1016/j.chest.2020.06.064 32634435

[7] Chen S, Gao G, Xia Y, Wu Z (2023) Incidence rate and risk factors of ventilator-associated pneumonia in patients with traumatic brain injury: a systematic review and meta-analysis of observational studies. J Thorac Dis. 15(4):2068 37197499 10.21037/jtd-23-425 PMC10183555

[8] Li Y et al (2020) Incidence, risk factors, and outcomes of ventilator-associated pneumonia in traumatic brain injury: a meta-analysis. Neurocrit Care 32(1):272–285 31300956 10.1007/s12028-019-00773-w PMC7223912

[9] Hu PJ et al (2017) Acute brain trauma, lung injury, and pneumonia: more than just altered mental status and decreased airway protection. Am J Physiol Lung Cell Mol Physiol 313(1):L1-l15 28408366 10.1152/ajplung.00485.2016

[10] Zaragoza R et al (2020) Update of the treatment of nosocomial pneumonia in the ICU. Crit Care 24(1):383 32600375 10.1186/s13054-020-03091-2 PMC7322703

[11] Chacón-Aponte AA et al (2022) Brain-lung interaction: a vicious cycle in traumatic brain injury. Acute Crit Care 37(1):35–44 35172526 10.4266/acc.2021.01193 PMC8918716

[12] Bergstrom N et al (1987) The Braden Scale for Predicting Pressure Sore Risk. Nurs Res 36(4):205–210 3299278

[13] Mehicic A, Burston A, Fulbrook P (2024) Psychometric properties of the Braden scale to assess pressure injury risk in intensive care: A systematic review. Intensive Crit Care Nurs 83:103686 38518454 10.1016/j.iccn.2024.103686

[14] Jia Y et al (2020) Prognostic value of braden scale in patients with acute myocardial infarction: from the retrospective multicenter study for early evaluation of acute chest pain. J Cardiovasc Nurs 35(6):E53-e61 32740222 10.1097/JCN.0000000000000735

[15] Tang Y, Li X, Cheng H, et al. (2025) Braden score predicts 30-day mortality risk in patients with ischaemic stroke in the ICU: a retrospective analysis based on the MIMIC-IV database. Nurs Crit Care 30(3):e13125. 10.1111/nicc.13125 10.1111/nicc.13125 39030917

[16] Cheng H et al (2024) Can admission Braden skin score predict delirium in older adults in the intensive care unit? Results from a multicenter study. J Clin Nurs 33(6):2209–2225 38071493 10.1111/jocn.16962

[17] Lovicu E, Faraone A, Fortini A (2021) admission braden scale score as an early independent predictor of in-hospital mortality among inpatients with COVID-19: a retrospective cohort study. Worldviews Evid Based Nurs 18(5):247–253 34275200 10.1111/wvn.12526 PMC8447426

[18] Jentzer JC et al (2019) Admission braden skin score independently predicts mortality in cardiac intensive care patients. Mayo Clin Proc 94(10):1994–2003 31585582 10.1016/j.mayocp.2019.04.038

[19] Ding Y et al (2019) Braden scale for assessing pneumonia after acute ischaemic stroke. BMC Geriatr 19(1):259 31590645 10.1186/s12877-019-1269-x PMC6781366

[20] Ding Y, Ji Z, Liu Y, Niu J (2022) Braden scale for predicting pneumonia after spontaneous intracerebral hemorrhage. Revista da Associação Médica Brasileira. 8(68):904–11 10.1590/1806-9282.20211339 PMC9574960 35946766

[21] Johnson AEW et al (2023) MIMIC-IV, a freely accessible electronic health record dataset. Sci Data 10(1):1 36596836 10.1038/s41597-022-01899-x PMC9810617

[22] Johnson AE et al (2016) MIMIC-III, a freely accessible critical care database. Sci Data 3:160035 27219127 10.1038/sdata.2016.35 PMC4878278

[23] Lu Y et al (2024) An externally validated prognostic model for critically ill patients with traumatic brain injury. Ann Clin Transl Neurol 11(9):2350–2359 38973122 10.1002/acn3.52148 PMC11537144

[24] Qi L et al (2024) Association of glycemic variability and prognosis in patients with traumatic brain injury: a retrospective study from the MIMIC-IV database. Diabetes Res Clin Pract 217:111869 39332533 10.1016/j.diabres.2024.111869

[25] Drahos J et al (2013) Accuracy of ICD-9-CM codes in identifying infections of pneumonia and herpes simplex virus in administrative data. Ann Epidemiol 23(5):291–293 23522903 10.1016/j.annepidem.2013.02.005 PMC3654522

[26] Warren C et al (2019) A Nurse-Driven Oral Care Protocol to Reduce Hospital-Acquired Pneumonia. Am J Nurs 119(2):44–51 30681478 10.1097/01.NAJ.0000553204.21342.01

[27] Yang R et al (2022) The use of antibiotics for ventilator-associated pneumonia in the MIMIC-IV Database. Front Pharmacol 13:869499 35770093 10.3389/fphar.2022.869499 PMC9234107

[28] Delmore BA, Ayello EA (2023) Braden scales for pressure injury risk assessment. Adv Skin Wound Care 36(6):332–335 37212567 10.1097/01.ASW.0000931808.23779.44

[29] Johnston R, Jones K, Manley D (2018) Confounding and collinearity in regression analysis: a cautionary tale and an alternative procedure, illustrated by studies of British voting behaviour. Qual Quant 52(4):1957–1976 29937587 10.1007/s11135-017-0584-6 PMC5993839

[30] Vrieze SI (2012) Model selection and psychological theory: a discussion of the differences between the Akaike information criterion (AIC) and the Bayesian information criterion (BIC). Psychol Methods 17(2):228–243 22309957 10.1037/a0027127 PMC3366160

[31] Torres A, Niederman MS, Chastre J, Ewig S, Fernandez-Vandellos P, Hanberger H, Kollef M, Bassi GL, Luna CM, Martin-Loeches I, Paiva JA. International ERS/ESICM/ESCMID/ALAT guidelines for the management of hospital-acquired pneumonia and ventilator-associated pneumonia: Guidelines for the management of hospital-acquired pneumonia (HAP)/ventilator-associated pneumonia (VAP) of the European Respiratory Society (ERS), European Society of Intensive Care Medicine (ESICM), European Society of Clinical Microbiology and Infectious Diseases (ESCMID) and Asociación Latinoamericana del Tórax (ALAT). European Respiratory Journal. 2017 Sep 10;50(3). 10.1183/13993003.00582-2017 10.1183/13993003.00582-2017 28890434

[32] Reizine F, Asehnoune K, Roquilly A, Laviolle B, Rousseau C, Arnouat M, Dahyot-Fizelier C, Seguin P (2019) Effects of antibiotic prophylaxis on ventilator-associated pneumonia in severe traumatic brain injury. A post hoc analysis of two trials. J Critic Care. 1(50):221–6 10.1016/j.jcrc.2018.12.010 30583121

[33] Brueske BS et al (2022) Braden skin score subdomains predict mortality among cardiac intensive care patients. Am J Med 135(6):730-736.e5 35202570 10.1016/j.amjmed.2022.01.046

[34] Nygaard H, Kamper RS, Nielsen FE, Hansen SK, Hansen P, Wejse MR, Pressel E, Rasmussen J, Suetta C, Ekmann A (2024) The hazard of mortality across different levels of frailty are increased among patients with high Braden scores. Eur Geriatr Med 15(6):1899–908 39342075 10.1007/s41999-024-01062-2 PMC11632018

[35] Li Z et al (2022) Relationship between Braden Scale scores and acute kidney injury among patients with acute coronary syndrome: a multicentre retrospective cohort study. BMJ Open 12(1):e049102 34987039 10.1136/bmjopen-2021-049102 PMC8734026

[36] Geng X et al (2023) Construction and validation of a predictive model of pneumonia for ICU patients with traumatic brain injury (TBI). Neurosurg Rev 46(1):308 37985473 10.1007/s10143-023-02208-9

[37] Wang R et al (2023) Machine learning algorithms for prediction of ventilator associated pneumonia in traumatic brain injury patients from the MIMIC-III database. Heart Lung 62:225–232 37595390 10.1016/j.hrtlng.2023.08.002

[38] Li S et al (2024) A machine learning model based on ct imaging metrics and clinical features to predict the risk of hospital-acquired pneumonia after traumatic brain injury. Infect Drug Resist 17:3863–3877 39253609 10.2147/IDR.S473825 PMC11382661

[39] Abujaber A, Fadlalla A, Gammoh D, Al-Thani H, El-Menyar A (2021) Machine learning model to predict ventilator associated pneumonia in patients with traumatic brain injury: the C 5 decision tree approach. Brain Injury. 35(9):1095–102 34357830 10.1080/02699052.2021.1959060

[40] Raj R et al (2014) Predicting six-month mortality of patients with traumatic brain injury: usefulness of common intensive care severity scores. Crit Care 18(2):R60 24708781 10.1186/cc13814 PMC4056363

[41] Koutsoukou A et al (2006) Respiratory mechanics in brain-damaged patients. Intensive Care Med 32(12):1947–1954 17053881 10.1007/s00134-006-0406-0

[42] Gonzalvo R et al (2007) Bench-to-bedside review: brain-lung interaction in the critically ill–a pending issue revisited. Crit Care 11(3):216 17581271 10.1186/cc5930 PMC2206421

