# Supplementary material for: Admission braden scale is an effective marker for predicting pneumonia in critically ill patients with traumatic brain injury
Source: Neurosurg Rev. 2025 May 21;48(1):434. doi: 10.1007/s10143-025-03571-5 (PMC12095439; doi:10.1007/s10143-025-03571-5)
Supplement: Supplementary file 1 — Supplementary file1 (DOCX 625 KB) [file 10143_2025_3571_MOESM1_ESM.docx]

**Admission Braden Scale is an effective marker for predicting pneumonia in critically ill patients with traumatic brain injury**

**Supplementary Materials**

**
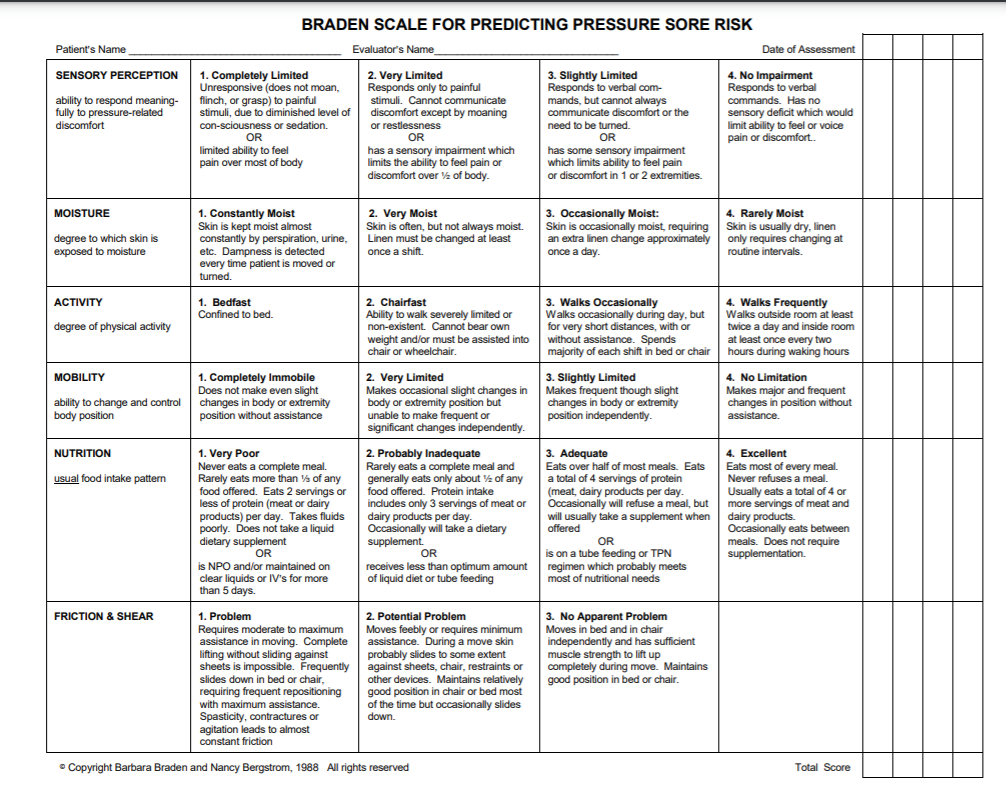
**

**Supplementary Figure 1**. The content of Braden scale.


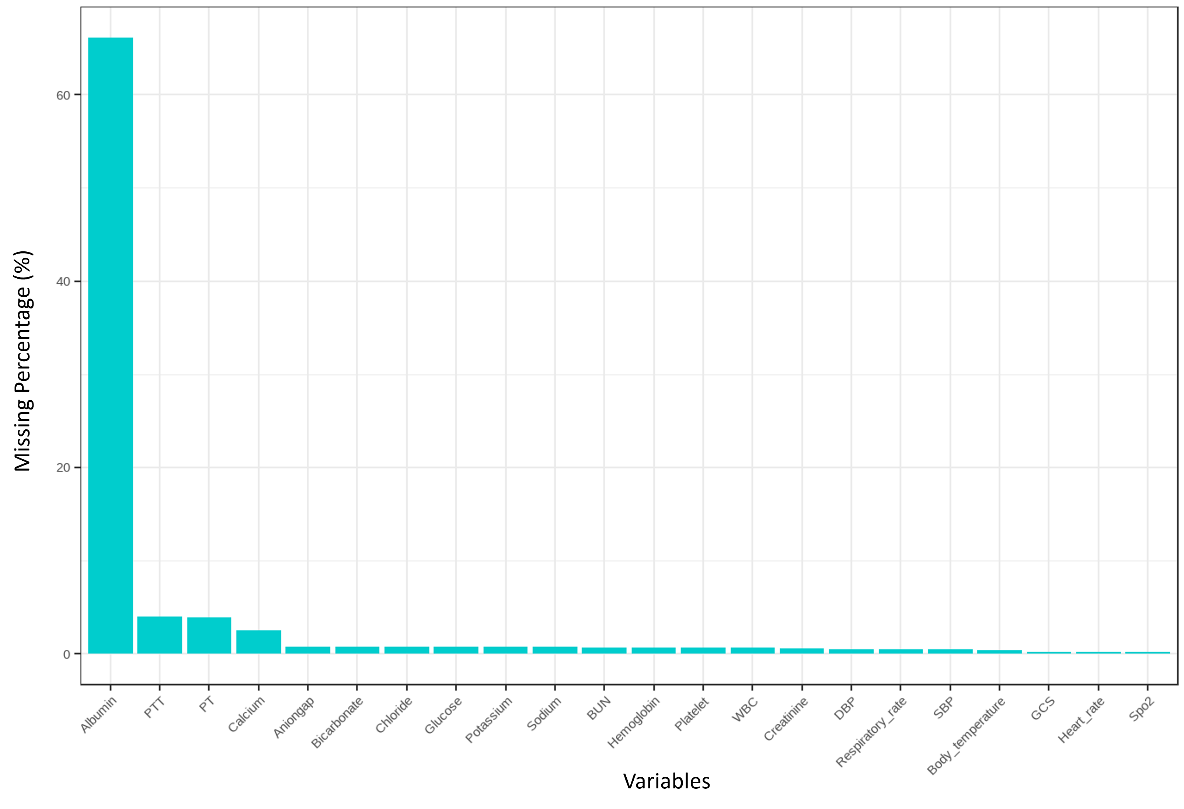


**Supplementary Figure 2**. The percentage of missing values (%) for variables in the final cohort. Variables with over 10% missing data were removed to ensure study accuracy. BUN blood urea nitrogen, DBP diastolic blood pressure, GCS Glasgow Coma Score, PT Prothrombin time, PTT partial thromboplastin time, SBP systolic blood pressure, SpO_2_ oxyhemoglobin saturation, WBC white blood cell.

**Supplementary Table 1.** Variance inflation factor for the risk factors in the nomogram

| **Variables** | **VIF** |
| --- | --- |
| Braden Scale | 1.3198 |
| Male | 1.2327 |
| Calcium | 1.4083 |
| Heart failure | 1.2128 |
| Chronic pulmonary disease | 1.1037 |
| Sepsis | 1.2286 |
| Respiratory rate | 1.2832 |
| Body Temperature | 1.2788 |

VIF Variance inflation factor.

**Supplementary Table 2.** A Comparative Analysis of the Predictive Value of BS, a model without BS, and the model incorporating BS

| **Variables** | **AUC**  **(95% CI)** | **P value** | **NRI**  **(95% CI)** | **P value** | **IDI**  **(95% CI)** | **P value** |
| --- | --- | --- | --- | --- | --- | --- |
| BS | 0.628  (0.605-0.652) | Ref. | - | - | - | - |
| Model without BS | 0.796  (0.775-0.817) | Ref. | Ref. | - | Ref. | - |
| Model with BS (the final model) | 0.803  (0.782-0.824) | **P_1_< 0.001**  **P_2_= 0.028** | 0.515 (0.420-0.609) | **<0.001** | 0.011  (0.006-0.016) | **<0.001** |

**Note:** P_1_: compared to BS; P_2_: compared to the model without BS. The final model includes predictors such as BS, male, calcium, heart failure, chronic pulmonary disease, sepsis, respiratory rate, and body temperature. Ref. reference, BS Braden Scale, AUC: area under the receiver operator characteristic curve, NRI: net reclassification improvement, IDI: integrated discrimination improvement.
